# Leveraging investments in Ebola preparedness for COVID-19 in Sub-Saharan Africa

**DOI:** 10.12688/aasopenres.13052.1

**Published:** 2020-03-18

**Authors:** Rodgers Ayebare, Peter Waitt, Stephen Okello, Mubaraka Kayiira, Maureen Atim Ajok, Irene Nakatudde, Nahid Bhadelia, Mohammed Lamorde

**Affiliations:** 1 Department of Global Health Security, Infectious Diseases Institute, Makerere University, Kampala, Kampala, 000256, Uganda; 2 Makerere University Walter Reed Porject, Kampala, Kampala, 000256, Uganda; 3 Emerging Infectious Diseases Laboratories, Section of Infectious Diseases, Boston University School of Medicine, Boston, Massachusetts, 02118, USA

**Keywords:** COVID-19 preparedness, Ebola, Coronavirus, SARS-CoV-2

## Abstract

The emergence of SARS-CoV-2 in China and transmission to more than 80 territories worldwide, including nine countries in Africa, presents a delicate situation for low-resource settings. Countries in Eastern and Central Africa have been on high alert since mid-2018 in anticipation of regional spread of the Ebola virus from the Democratic Republic of Congo. Significant investment has been made to support enhanced surveillance at point of entry and hospitals, infection control practices, clinical case management, and clinical research. With a new threat on the horizon, African countries have an opportunity to leverage the existing capacities for Ebola preparedness to brace for the imminent threat.

## Background

A novel Coronavirus (SARS-CoV-2) rapidly emerged in China and has spread internationally. On Jan 30, 2020, it was declared a Public Health Emergency of International Concern (PHEIC) by the World Health Organization (WHO) after exceeding 10,000 cases and 200 deaths, with 18 countries reporting cases. The declaration was in part justified by the need to strengthen preparedness in countries with weaker health systems ^1^. Concerns exist about these countries’ capacity to prevent, detect and respond to the COVID-19 outbreak. As of the time of writing, more than 30 African countries with 25 in sub-Saharan Africa have each reported a case of COVID-19 and 18 other countries are at risk of importation of a COVID-19 case from China. Weaknesses in public health systems were a prominent driver of the 2013–2016 Ebola virus disease (EVD) outbreaks in West Africa and similar challenges have fuelled the 2018 EVD outbreak in the Democratic Republic of the Congo (DRC), which is ongoing. We, therefore, questioned whether capacities enhanced for EVD could be leveraged to SARS-CoV-2, a biologically distinct virus requiring a broader public health response.

## Coordination

Coordination structures are essential to ensure emergency and contingency plans are in place, operational structures exist with clear communication channels, and adequate resources are available for impending threats. During the West Africa EVD outbreak, Nigeria, transitioned its emergency coordination centres and public health activities for polio eradication to respond to EVD ^2^. In the current EVD outbreak in DRC, the WHO has provided dedicated preparedness support to enhance national capacities for EVD in DRC and its nine neighbouring countries. Currently, seven out of the 10 countries have met their minimum targets for EVD coordination ^3^. The declaration of a PHEIC is a timely intervention to enable African countries to mobilize resources domestically and through international sources to operationalise preparedness plans. Utilization of existing structures will be critical for the timely organization of preparedness and response efforts. Yields from this resource have been key in establishing SARS-CoV-2 testing capacity in over 40 countries on the continent within one month of declaration of a PHEIC through the Africa CDC coordination body.

## Risk communication

Risk communication entailing significant community outreach and education on infection prevention and control as part of the EVD response has supported improvements in hand hygiene, social distancing, case identification and reporting. Similarly, risk communication is needed for COVID-19 ensure standard precautions are enhanced particularly in the context of respiratory hygiene. To date, the risk communication platforms are being utilized to disseminate infection control measures for COVID-19 and to identify public myths about the novel disease condition, so that targeted communication to demystify public confusion and rumours is delivered.

## Personal protective equipment logistics

In healthcare settings, careful logistics planning is critical to ensure panic buying of face masks and respirators by the general public does not lead to scarcity in health units. Stockpiles of personal protective equipment for EVD could in the interim, support some needs for COVID-19 but such decisions should only be made after careful assessment of ongoing risk of importation of EVD. Unfortunately, these same countries must also plan for scenarios with concurrent outbreaks for COVID-19 and EVD and test their systems to ensure resilience against resource limitations and workforce fatigue.

## Surveillance

Surveillance efforts deployed for EVD in Eastern and Central Africa could be modified to incorporate current case definitions for COVID-19. Consequently, surveillance in health facilities will be critical, including in private facilities frequented by international travellers and intensive care units that may not have been adequately addressed in EVD preparedness efforts. Already, expensive screening for EVD at land borders neighbouring DRC could require an extension to all national borders if COVID-19 cases are reported in surrounding countries. In countries with laboratory detection capacity, testing is currently centralised in a few laboratories that meet the necessary biosecurity requirements. While domestic and international efforts are underway to acquire more testing capacity, sample collection and transportation systems enhanced for EVD and international referral diagnostic testing could be utilized to inform public health and clinical management strategies.

## Infection control, clinical case management capabilities and use of investigational therapeutics

Biological differences between Ebola virus and SARS-CoV-2 in the mode of transmission and case presentation will limit some benefits of EVD preparedness. Country scenarios must include plans for exponentially larger patient numbers than for EVD. While EVD requires close contact, COVID-19 is transmitted mainly through droplets, contact with contaminated hands and potentially through aerosol-forming procedures. Facility-based isolation capacity is likely to be exceeded during a large outbreak and self-quarantine at home may be needed for milder cases. Cohorting severe cases under investigation will be highly dependent on the availability of appropriate hospital beds, laboratory confirmation capacity and a skilled workforce. Hospital-based clinical case management teams that have received training in care for critically ill patients are a resource to leverage to bridge the gap for COVID-19. In Uganda, the existence five teams based at five different hospitals, two of which are in the capital, is an example of a scalable baseline human resource capacity. Cardinal features of the training such teams have received include infection prevention and control ^4^, outbreak investigation, laboratory, clinical case management.

Optimised supportive clinical care for EVD patients has been recently introduced but will be challenging to scale, even if adapted for COVID-19. Respiratory support in dedicated facilities may rapidly become inadequate and consequently, efforts to detect early and contain imported cases are critical. African countries can engage in research for medical countermeasures (vaccines and experimental drugs). The successful conduct of the PALM trial (PAmoja tuLinde Maisha) in the ongoing EVD outbreak in DRC serves as an example that can be used for therapeutic research for COVID-19 ^5^. Sub-Saharan Africa also has vast experience working with some of the investigational products being studied in China; ritonavir-boosted lopinavir has been used in Sub-Saharan Africa as antiretroviral therapy for treatment-experienced patients in Sub-Saharan Africa and most recently, remdesivir was used in the PALM trial for EVD in DRC. With results from the COVID-19 trials set to be made available soon, Africa has a unique opportunity to rapidly access life-saving investigational therapeutics that are familiar and potentially readily available.

## Conclusion

Although challenges remain, African countries that have been supported for EVD preparedness in ongoing and past EVD outbreaks have capacities that can be enhanced for the COVID-19 preparedness and response.

## Data availability

No data are associated with this article.

## References

[1] WHO: Statement on the second meeting of the International Health Regulations (2005) Emergency Committee regarding the outbreak of novel coronavirus (2019-nCoV).30 January 2020 Statement, Geneva, Switzerland. Reference Source

[2] Rutter PD Hinman AR Hegg L: Transition Planning For After Polio Eradication. *J Infect Dis.* 2017;216(suppl_ 1):S287–S292. 10.1093/infdis/jix026 28838183 PMC5853549

[3] World Health Organization: Ebola Operational Readiness and Preparedness - Preparedness Map. (Accessed on 02 Feb 2020). Reference Source

[4] Biedron C Lyman M Stuckey MJ: Evaluation of Infection Prevention and Control Readiness at Frontline Health Care Facilities in High-Risk Districts Bordering Ebola Virus Disease-Affected Areas in the Democratic Republic of the Congo - Uganda, 2018. *MMWR Morb Mortal Wkly Rep.* 2019;68(39):851–854. 10.15585/mmwr.mm6839a4 31581162 PMC6776373

[5] Mulangu S Dodd LE Davey RT Jr: A randomized, controlled trial of ebola virus disease therapeutics. *N Engl J Med.* 2019;381(24):2293–2303. 10.1056/NEJMoa1910993 31774950 PMC10680050

