# Supplementary material for: Leveraging investments in Ebola preparedness for COVID-19 in Sub-Saharan Africa
Source: AAS Open Res. 2020 Mar 18;3:3. [Version 1] doi: 10.12688/aasopenres.13052.1 (PMC7236423; doi:10.12688/aasopenres.13052.1)
Supplement: Peer review history

# Peer review: Reviewer response for version 1

**DOI:** 10.21956/aasopenres.14147.r27445

**Published:** 2020-05-27

**Authors:** Christopher J. Morgan

**Affiliations:** 1 Burnet Institute, Melbourne, Vic, Australia

This concise, well-expressed open letter addresses the highly relevant topic of commonalities in emergency responses for immunization, EVD and COVID-19 and their importance to health system strength and resilience. This reviewer notes anecdotal reports of similar pivoting to COVID-19 by coordination systems for emergency responses to polio in Papua New Guinea. This letter is timely and well presented.

Minor suggestions include:

 Coordination:

- Make specific mention of the **rapid response and contract tracing functions** under polio emergency responses, as applied first to EVD and now to COVID-19;
- Consider whether new communication channels such as WhatsApp group necessitated by travel restrictions, apply as examples in this analysis.

Surveillance:

- Consider whether the option of integrated surveillance for vaccine-preventable-disease syndromes (e.g. measles) and for other public health priorities apply to this analysis, noting the likely decline in essential health service coverage will increase vulnerability to other outbreaks.
- Consider including mention of rapid diagnostics and low-cost sample transfer, which may become available and differ for COVID-19 from EVD.

Infection control:

- Given the dynamic nature of COVID-19 evidence, consider review and update of modes of spread.
- Use of "Cohorting..." as verb slightly confusing, consider revision - also seems to repeat idea in preceding sentence.
- Minor grammatical error in sentence "In Uganda, the existence ...".
- Consider special mention of oxygen (by concentrator or cylinder supply) as an essential medicine, with or without assisted ventilation, as a distinctive need of COVID-19 at all levels of clinical care (see open letter from stoppneumonia.org).

Overall, noting over 400 views now, congratulate the authors on this submission.

https://stoppneumonia.org/open-letter-to-leaders-of-the-global-coronavirus-response-on-access-to-medical-oxygen/

Does the article adequately reference differing views and opinions?

Yes

Are all factual statements correct, and are statements and arguments made adequately supported by citations?

Yes

Is the Open Letter written in accessible language?

Yes

Where applicable, are recommendations and next steps explained clearly for others to follow?

Partly

Is the rationale for the Open Letter provided in sufficient detail?

Yes

Reviewer Expertise:

Global immunization, health services delivery in resource-constrained settings, global child public health.

I confirm that I have read this submission and believe that I have an appropriate level of expertise to confirm that it is of an acceptable scientific standard.

# Peer review: Reviewer response for version 1

**DOI:** 10.21956/aasopenres.14147.r27446

**Published:** 2020-05-18

**Authors:** Molefe B. Phirinyane

**Affiliations:** 1 Botswana Institute for Development Policy Analysis (BIDPA), Gaborone, Botswana

The letter is written in clear language and a format accessible for decision makers and clinical practitioners. A few comments to make: 

1. While the authors duly note the distinct biological characteristics of SARS-COV-2 from EVD, the article could benefit from the acknowledgement that researchers worldwide are still learning the effects of COVID-19 on patients, such as organ failure, apart from respiratory problems. It would be interesting what the implications of that would be on the clinical capabilities of African states to address the challenge. 
2. Sub-Saharan African countries often have financial challenges to effectively implement their health policies and other interventions. The authors could look into what fiscal lessons has Africa learnt from EVD that they could apply to COVID-19?
3. The EVD, as the authors state, affects largely two blocks of countries - central and west Africa. At that scale it would catch the attention of the African Union and trigger their response. This could have been brought on to bear on the collective response of the African countries under the auspices of the AU or sub-regional groups and the lessons this carries for their response to COVID-19.
4. This is a must read for all actors in the health sector in Sub-Saharan Africa. The piece is succinct and proposes pragmatic approaches African countries could explore. 

Does the article adequately reference differing views and opinions?

Yes

Are all factual statements correct, and are statements and arguments made adequately supported by citations?

Yes

Is the Open Letter written in accessible language?

Yes

Where applicable, are recommendations and next steps explained clearly for others to follow?

Yes

Is the rationale for the Open Letter provided in sufficient detail?

Yes

Reviewer Expertise:

Public policy, health governance, governance, decentralisation.

I confirm that I have read this submission and believe that I have an appropriate level of expertise to confirm that it is of an acceptable scientific standard.

# Peer review: Reviewer response for version 1

**DOI:** 10.21956/aasopenres.14147.r27403

**Published:** 2020-04-22

**Authors:** Gavin Yamey

**Affiliations:** 1 Center for Policy Impact in Global Health, Duke Global Health Institute, Duke University, Durham, NC, USA

Many thanks for asking me to review this very valuable open letter that describes how Ebola response and preparedness "assets" could be leveraged in tackling the current COVID-19 pandemic.

 I see that the letter has already been viewed over 300 times, which I believe attests to its value.

 The letter is short, beautifully written, clear, and concise. There has been much discussion and debate about both the strengths and challenges that countries in sub-Saharan Africa (SSA) face in preparing for and responding to COVID-19 - and this letter adds helpful information to the debate.

 I offer a small number of comments and responses:

1. Obviously the pandemic is evolving rapidly, and if you are producing a version 2, it would be good to update the introductory situation analysis on SSA (https://covid19.who.int/).
2. Under coordination, the authors say “Yields from this resource have been key” - but it is not clear what "this resource" refers to.
3. I think it would be helpful to briefly discuss the role of the Africa CDC, which was launched in the wake of the 2014-2016 Ebola epidemic. It is playing a role not just in coordination but in communication, public health messaging, and other activities (https://africacdc.org/covid-19/).
4. Under risk communication, it is hard to grasp what this means: "“risk communication is needed for COVID-19 ensure standard precautions.”
5. Under risk communication, I think it would be very helpful to briefly describe how or whether there are lessons from Ebola for how to tailor messaging to different settings. For example, there is much discussion right now about the feasibility of social distancing among the informal labor force, or in camps for internally displaced persons.
6. Under case management, it would be good to add a sentence or two on options for respiratory support where there are insufficient ventilators (what are the alternatives?).
7. The letter states that "African countries can engage in research for medical countermeasures (vaccines and experimental drugs)." I think diagnostics should also be included as a MCM.

Does the article adequately reference differing views and opinions?

Yes

Are all factual statements correct, and are statements and arguments made adequately supported by citations?

Yes

Is the Open Letter written in accessible language?

Yes

Where applicable, are recommendations and next steps explained clearly for others to follow?

Partly

Is the rationale for the Open Letter provided in sufficient detail?

Yes

Reviewer Expertise:

Global health and public policy

I confirm that I have read this submission and believe that I have an appropriate level of expertise to confirm that it is of an acceptable scientific standard.
